# Leishmaniasis: A new method for confirming cure and detecting asymptomatic infection in patients receiving immunosuppressive treatment for autoimmune disease

**DOI:** 10.1371/journal.pntd.0009662

**Published:** 2021-08-02

**Authors:** Laura Botana, Ana Victoria Ibarra-Meneses, Carmen Sanchez, Belen Matia, Juan Victor San Martin, Javier Moreno, Eugenia Carrillo

**Affiliations:** 1 WHO Collaborating Centre for Leishmaniasis, National Centre for Microbiology, Instituto de Salud Carlos III, Majadahonda (Madrid), Spain; 2 Cardiology Department, Universidad Francisco de Vitoria/Hospital Ramón y Cajal Research Unit (IRYCIS), Madrid, Spain; 3 Hospital Universitario de Fuenlabrada, Fuenlabrada, Madrid, Spain; Centro de Pesquisa Gonçalo Moniz-FIOCRUZ/BA, BRAZIL

## Abstract

Visceral leishmaniasis (VL) in patients receiving immunosuppressant drugs for autoimmune disease has been on the rise. It is important—but difficult—to know when cure has been achieved in these patients since the withdrawal of immunosuppressants during antileishmania treatment is commonly required, and there is a risk of relapse when immunosuppression is restored. The prevalence of asymptomatic infection among those immunosuppressed for autoimmune disease is also uncertain. The present work describes how cytokine release assays can be used to confirm the cure of VL, and to determine the prevalence of asymptomatic infection, in such patients. After collection of blood from volunteers (n = 108), SLA-stimulation of peripheral blood mononuclear cell cultures and of whole blood was found to induce the production of different combinations of cytokines that served to confirm recovery from VL, and asymptomatic *Leishmania* infection. Indeed, cure was confirmed in 14 patients, all of whom showed a specific Th1 immune response against *Leishmania*, and the prevalence of asymptomatic infection was determined as 21.27%. Cytokine profiles could be used to manage VL in patients with autoimmune disease, and to identify and better protect those with asymptomatic infection who are at risk of developing this disease.

## Author summary

Cytokine release assays were used to confirm the cure of visceral leishmaniasis in patients (from an area where *Leishmania infantum* is endemic) with autoimmune disease. They were also used to determine the prevalence of asymptomatic *Leishmania infantum* infection in similar patients. Some 21.27% of those with no clinical history of leishmaniasis were shown to have been contact with the parasite.

## Introduction

Leishmaniasis, caused by *Leishmania infantum*, is endemic throughout peninsular Spain and the Balearic Islands [1]. The annual incidence of this disease is 0.45/100,000 people, although during an outbreak in Fuenlabrada (southeast of the Madrid region) in 2009 it reached 21.54/100,000 [2].

Immunosuppression is a major risk factor for the development of the disease; it can also alter how it presents and the response to treatment [3]. The focus on this problem has mainly been within the context of HIV, but treatment for autoimmune conditions with immunosuppressive drugs is becoming more common [4,5], leaving patients more vulnerable to leishmaniasis. For example, the appearance of visceral leishmaniasis (VL) has been associated with the use of different immunosuppressants administered in the treatment of rheumatism, including methotrexate, steroids and cyclosporin [6]. It is important—but difficult—to know when cure has been achieved in these patients since the withdrawal of immunosuppressants during antileishmania treatment is commonly required, and there is a risk of relapse when immunosuppression is restored. In addition, in *Leishmania*-endemic areas, relatively high case numbers of VL have been noticed among people treated with tumour necrosis factor (TNF) antagonists or blockers of the TNF receptor for several months [7]. Very little is known, however, about the relationship between leishmaniasis and pharmacological immunosuppression. Nor is the prevalence of asymptomatic *Leishmania* infection in this immunosuppressed population known.

A lymphoproliferative response to soluble *Leishmania* antigen (SLA), along with the production of cytokines associated with the Th1 response, is a marker of recovery from VL and a sign that immune memory against the parasite has been achieved [8]. Cytokine release assays are also now beginning to be used to identify asymptomatic patients [9–12]. The present work describes how these assays can be used to confirm the cure of VL in patients with autoimmune disease, and to determine the prevalence of asymptomatic *Leishmania* infection in the population receiving immunosuppressants. This method could be used better manage VL in patients with autoimmune disease—in whom immunosuppression is normally halted during antiparasite treatment—and to identify patients with autoimmune disease who have asymptomatic *Leishmania* infection, who are at risk of developing VL because of their immunosuppressive treatment. To our knowledge, this is the first work to study cytokine production in such patients.

## Materials and methods

### Ethics statement

This study was approved by the *Hospital de Fuenlabrada* (APR 12–65 and APR14-64). All participants gave their written, informed consent to be included.

### Patients and blood samples

Blood samples were taken from 108 volunteers undergoing immunosuppressant treatment for autoimmune conditions at the above hospital, all of whom were aged over 18 years. All were residents of Fuenlabrada (Madrid, Spain), an area endemic for *Leishmania infantum* and where the overall prevalence of infection is high. Of these 108 patients, 94 had no history of leishmaniasis (NH), and 14 were ostensibly cured of VL (CVL patients) according to WHO criteria (i.e., no symptoms 6 months after completing pharmacological anti-*Leishmania* treatment; blood samples for these patients were taken 6 months after treatment for VL ended). Blood samples were also available for 5 of these latter 14 patients during the time when they had active VL (AVL). All blood samples were subjected to specific humoral, cellular and molecular tests for *Leishmania*, and their cytokine profiles determined. Cellular tests were always performed within 24 hours after sample reception.

The case definition of AVL was clinical manifestations compatible with the condition plus at least one of the following [7]: (i) positive parasitological test (blood/bone marrow PCR); and/or (ii) positive serological [rK39-ICT and ELISA/IFAT (enzyme-linked immunosorbent assay/indirect immunofluorescent antibody test)] test plus clinical response to treatment.

### Treatments administered

S1 and S2 Tables show the characteristics of, and treatments received by, the 94 NH patients and the 14 patients ostensibly cured of VL, respectively.

### Preparation of soluble *Leishmania* antigen

Soluble *L*. *infantum* antigen (SLA) was prepared from promastigote cultures in the stationary phase (JPC strain, MCAN/ES/98/LLM-722), as previously described [13]. The parasites were first washed with 1X phosphate-buffered saline (PBS) and centrifuged at 1000 g for 20 min at 4°C. The supernatant was discarded and the pellet resuspended in lysis buffer (50 mM Tris/5 mM EDTA/HCl, pH 7). These samples were subjected to three cycles of freezing/thawing, and then sonicated three times (40 W for 20 s). The parasite suspension was then centrifuged at 27,000 × *g* for 20 min. The supernatant was collected and centrifuged at 100,000 × *g* for 4 h, divided into aliquots, and stored at -80°C until use. The protein content was quantified following the Bradford method, using the Pierce BCA Protein Assay Kit (Thermo Fisher Scientific).

### Enzyme-linked immunosorbent assay

Enzyme-linked immunosorbent assay (ELISA) was used to detect antibodies in blood to SLA [12]. Briefly, 96-well plates (NuncMaxisorp Immuno Plates, USA) were coated with 100 μl/well of 10 μg/ml SLA and left overnight at 4°C. The plates were then washed three times with PBS, 0.1% Tween 20 (PBS-T), pH7.4, and blocked with 200 μl/well of PBS containing 0.1% Tween 20 and 3% BSA for 1 h at 37°C. After washing with PBS-T, diluted blood plasma (1/200 in PBS-T) was added (100 μl/well) and incubated for 2 h at 37°C. The plates were then washed with PBS-T and 100 μl/well of 1/5000-diluted HRP-conjugated anti-human Ig (Invitrogen, USA) were added for 30 min at 37°C. All plates were then developed with 100 μl/well of Sigma Fast o-phenylene diamine dihydrochloride (OPD) tablets (Sigma, USA) for 20 min. The reaction was stopped with 50 μl/well of 2NHCl, and absorbance measured at 492 nm.

### Immunofluorescent antibody titres

Immunofluorescent antibody titre (IFAT) analyses of plasma samples were performed using 2 × 10^5^ *L*. *infantum* promastigotes in PBS per well (MCAN/ES/98/LLM-722), as previously described [12]. Patient plasma was assayed as two-fold serial dilutions (from 1/20 to 1/640) in PBS to determine total IgG levels using fluorescein isothiocyanate-conjugated goat antihuman IgG (Fluoline G) (BioMérieux, France) diluted 1/200. The threshold titre for positivity was set at 1:80.

### rK39-ICT serological test

The rK39-ICT test (Leti Laboratories, Spain) is a rapid, commercial, immunochromatographic test for the quantitative detection of *Leishmania* antibodies in serum. Serum (25 μl) was added to the test strips, along with the provided buffer solution, in 2 ml Eppendorf tubes. After 10 min at room temperature, the strips were examined for the two bands (control and specific) indicating a positive result.

### DNA extraction and *Leishmania* nested PCR

DNA was extracted from 200 μl whole blood to which had been added 400 μl of NET10 (10 mM NaCl, 10 mM EDTA, 10 mM Tris HCl), 40 μl of SDS sample buffer (10%), and 2 μl of proteinase K. Samples were incubated with agitation overnight at 56°C. The DNA was isolated using the phenol-chloroform method, precipitating in ethanol [14]. The total DNA was resuspended in 100 μl of sterile distilled water and quantified using a UV-V ND-100 spectrophotometer (NanoDrop Technology, USA). The extracted DNA was subjected to nested PCR (Ln-PCR) using primer pairs that amplify the *Leishmania* small ribosomal subunit (SSUrRNA) [15], employing a GenAmp PCR System 2700 thermocycler (Applied Biosystems, USA). The first round of reactions (30 cycles, annealing temperature 60°C) involved the use of primers R221 and R332. The amplicons were diluted 1/40 in distilled water, and 10 μl of this dilution used in the second round of reactions, which involved the use of primers R223 and R333 (30 cycles, annealing temperature 65°C). The amplicons were then visualised in 1.5% agarose gels in TAE buffer (Tris-acetate 0.04 mM, EDTA 1 μM, pH 8) using 0.02% GelRed staining (Biotium, USA) under a MiniBis-proilluminator (DNR, Bio-imaging systems, Israel). Positive results require amplicons of 358 bp be detected.

### Culturing and stimulation of peripheral blood mononuclear cells (lymphoproliferative or cell proliferation assay [CPA])

Blood samples (10 ml) were collected in heparinised vials from all subjects. Peripheral blood mononuclear cells (PBMC) were separated out using a Ficoll-Hypaque gradient (Rafer, Spain), resuspended in complete RPMI supplemented with 10% foetal bovine serum, and cultured (in triplicate) at an initial concentration of 2x10^6 cells/ml in 96-well plates with either complete RPMI (negative control) or SLA (10 μg/ml) [12]. All cultures were kept for 6 days at 37°C in a 5% CO_2_ atmosphere. The lymphoproliferative response of each subject was then determined by bromodeoxyuridine incorporation using the Cell Proliferation Kit (GE Healthcare Life Sciences, UK), following the manufacturer’s instructions. Results were expressed in the form of a stimulation index (SI, the absorbance of stimulated cells/unstimulated cells). The cut-off for a positive lymphoproliferative response (stimulation index 2.5) was determined using a receiver operating curve (ROC). Note that the culture supernatants were collected and stored at -20°C for later cytokine analysis (see below).

### Stimulation of whole blood with SLA (whole blood assay [WBA])

Aliquots (500 μl) of whole blood were incubated in tubes with 10 μg/ml SLA or 5 μg/ml PHA-M. A further tube with no SLA was used as a negative control. All tubes were incubated at 37°C for 24 h, as previously described [10,12]. They were then centrifuged at 2000 *g* for 10 min. The supernatants were then removed and kept at -20°C for later cytokine analysis. Results for each cytokine were expressed as the difference between the SLA-stimulated and control plasma concentrations.

### Cytokine release assays

IL-2, IP-10, IFN-γ, TNF-α and granzyme B were determined in 50 μl of plasma from SLA-stimulated whole blood and the supernatants of SLA-stimulated PBMC (see above) using the CBA Human Soluble Protein Flex Set Capture Bead Kit (Becton Dickinson Spain) according to the manufacturer’s instructions. The flow cytometry results obtained were analysed using FCAP Array v.3.0.1 software. The cut-off for asymptomatic infection and cure for each cytokine were determined using receiver operating curves (ROC).

### Statistical analysis

The normality of distributions was checked using the Shapiro-Wilk test. Accordingly, the Mann-Whitney U test was used to analyse differences between the different groups. Significance was set at p<0.050. All calculations were made using GraphPad Prism v. 5 software.

## Results

### Immunological response and parasite load

Of the 108 patients examined, the 14 CVL patients all returned a negative Ln-PCR test and all mounted positive serological responses. Three of the five patients for whom blood was available before cure, i.e., when they still had AVL, returned a positive Ln-PCR test and at least one positive serological test (Table 1). The 94 NH patients returned negative serological and Ln-PCR tests (Table 1).

**Table 1:** Mean age of subjects and percentages with positive Ln-PCR, IFAT, rk39-ICT and ELISA tests.

	Age in years (mean ± SD)	% Ln-PCR positive	% IFAT positive	% rk39-ICT positive	% ELISA positive
**NH (n = 94)**	48 ± 13	0 (0/94)	0 (0/94)	0 (0/94)	0 (0/94)
**CVL (n = 14)**	59 ± 13	0 (0/14)	64 (9/14)	71 (10/14)	71 (10/14)
**AVL (n = 5)**	57 ± 12	60 (3/5)	100 (5/5)	80 (4/5)	100 (5/5)

**Fig 1:**
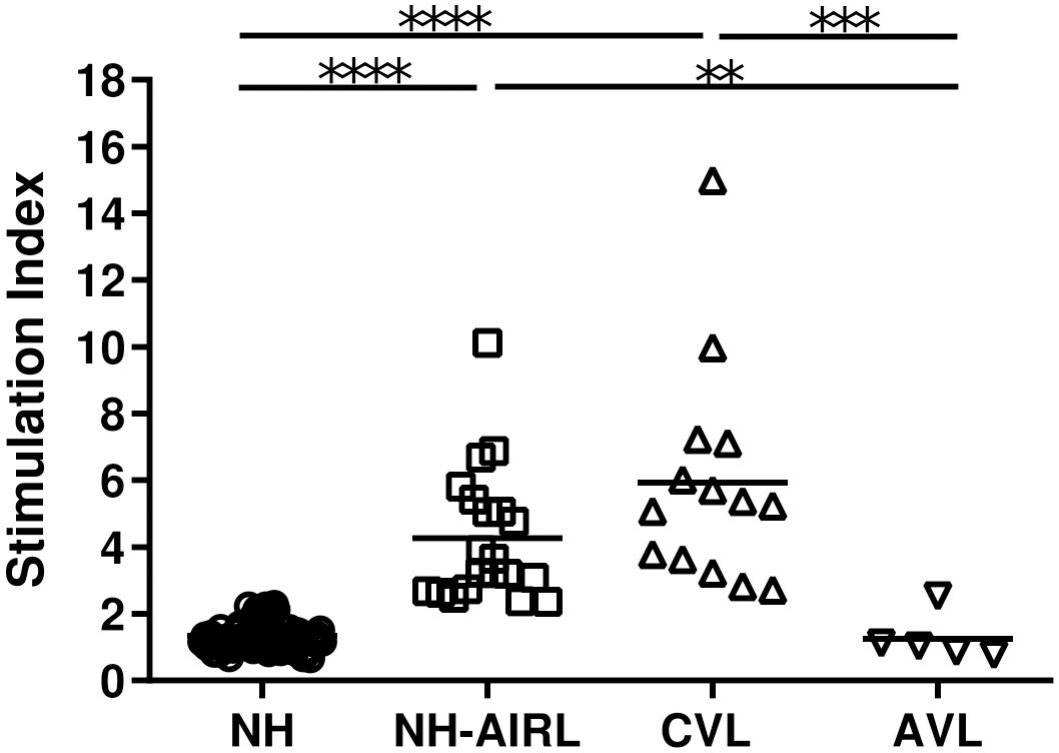
Lymphoproliferative response (CPA test) results following stimulation of their PBMC with SLA. Patients with no history of leishmaniasis who did not respond (*NH* n = 74), asymptomatic immune responders to *Leishmania* (*NH-AIRL* n = 20), patients cured of VL (*CVL* n = 14), and the five *AVL* patients (i.e., samples from 5 of the CVL patients during early disease). *p<0.050, ** p<0.010, *** p<0.001, **** p<0.0001.

Of the 94 NH subjects, 20 showed a positive CPA test under SLA challenge (p<0.0001); these were classified as asymptomatic immune responders to *Leishmania* (NH-AIRL) (Fig 1). The mean SI of these 20 subjects was 4.26 compared to 1.33 for the remaining 74 NH non-responders. The prevalence of asymptomatic infection was therefore 21.27% (20/94). All of the CVL patients returned a positive response (Fig 1), and showed a significantly different SI to the NH patients (p<0.0001). The five AVL patients all returned a negative result, with significant differences in SI with respect to the above NH-AIRL individuals and the CVL patients (p<0.010 and p<0.001 respectively).

Following stimulation of the PBMC of all subjects with PHA-M, no significant difference was seen among the NH [SI 6.422 (4.79–9.10)], NH-AIRL [SI 4.306 (3.55–8.91)], CVL [SI 9.93 (3.70–8.72)], and AVL [SI 3.739 (2.68–12.23)] groups in terms of response; they were well capable of responding to PHA-M.

### Cytokine profiles of supernatants from SLA-stimulated PBMC cultures

For practical and economic reasons, a random sample of 44 NH patients (hereinafter NC [normal control]) was selected from the original 74 NH non-responders, and their data used in the analysis of the cytokine profile results.

**Fig 2:**
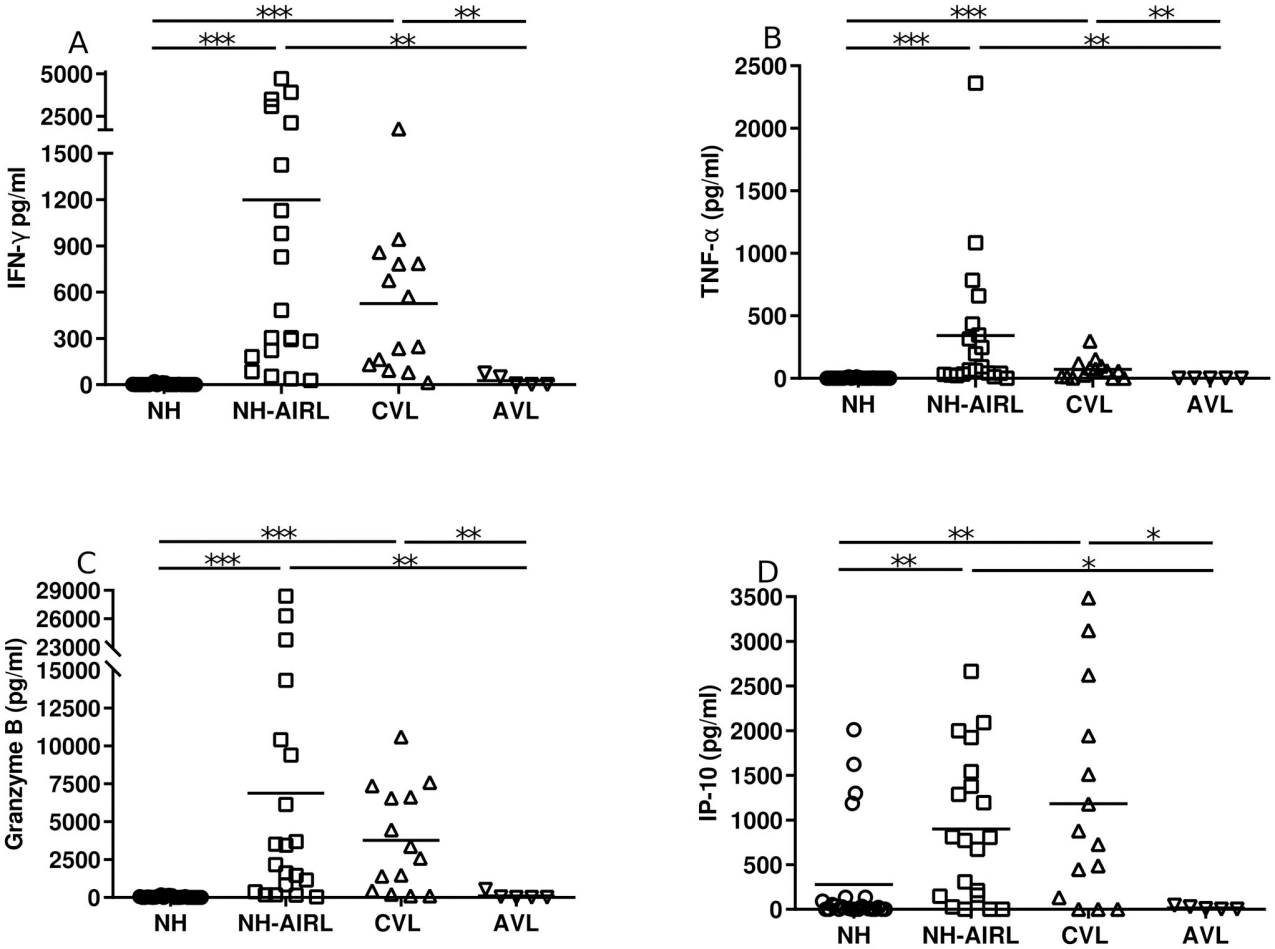
Production of a) IFN-γ, b) TNF-α, c) granzyme B and d) IP-10 in supernatants of PBMC cultures stimulated with SLA. *NC* normal controls, *NH-AIRL* asymptomatic immune responders to *Leishmania*, *CVL* patients cured of leishmaniasis, and *AVL* patients with active VL. *p<0.050, **p<0.010, *** p<0.001.

Compared to the NC patients, the NH-AIRL patients showed significantly higher values for IFN-γ (p<0.001), TNF-α (p<0.001), granzyme B (p<0.0005) and IP-10 (p<0.010) (Fig 2). The CVL patients had the same cytokine profile as the NH-AIRL patients, with no significant differences between these groups for any of the measured variables.

Compared to the CVL patients, the AVL patients returned significantly lower values for all four cytokines measured: IFN-γ (p<0.010), TNF-α (p<0.010), granzyme B (p< 0.010) and IP-10 (p<0.050) (Fig 2).

### Quantification of cytokines in plasma from SLA-stimulated whole blood

**Fig 3:**
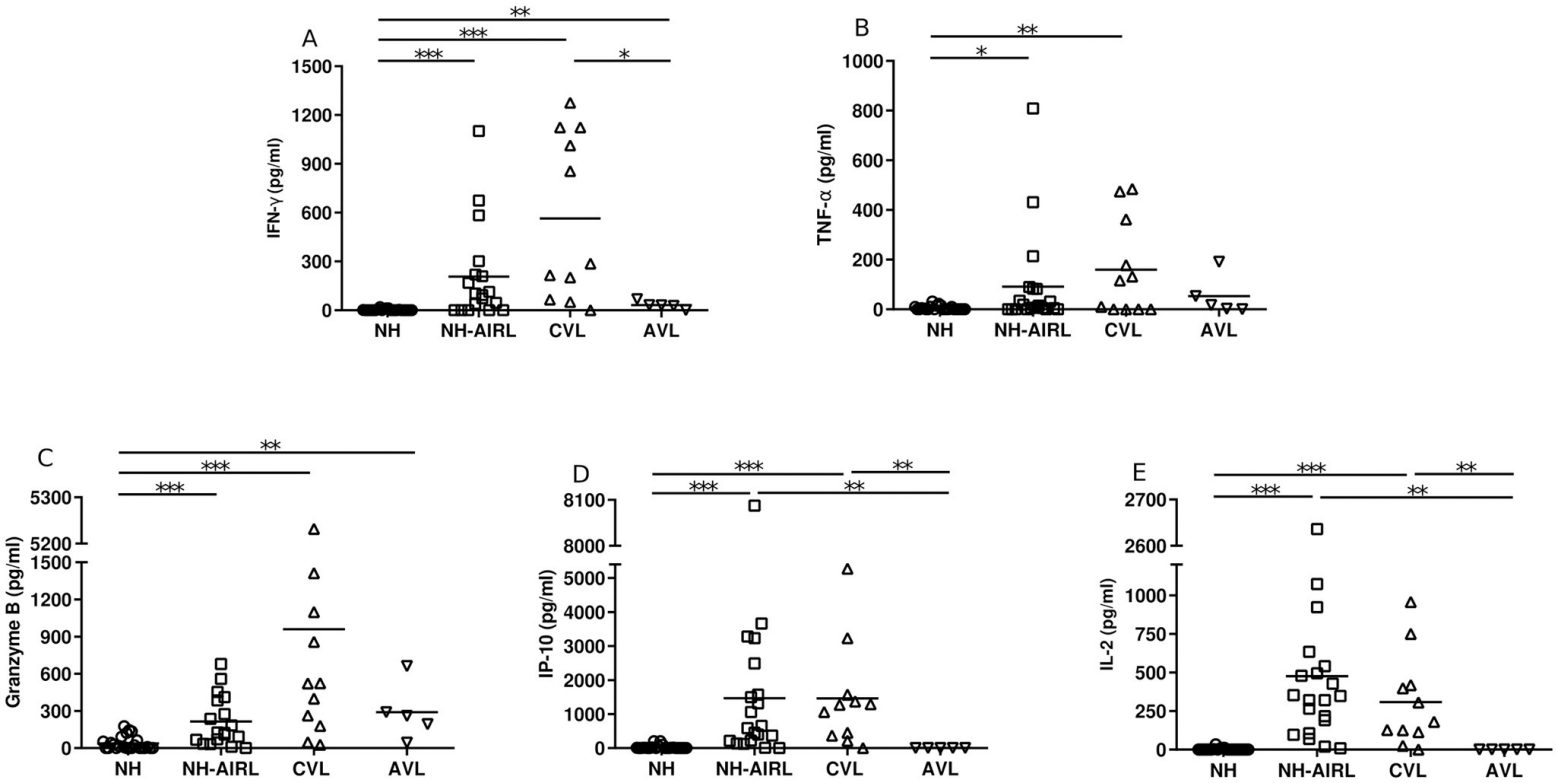
a) IFN-γ, b) TNF-α, c) granzyme B, d) IP-10 and e) IL-2 in whole blood stimulated with SLA. *NC* normal controls, *NH-AIRL* asymptomatic immune responders to *Leishmania*, *CVL* patients cured of leishmaniasis, and *AVL* patients with active VL. *p<0.050, **p<0.010, *** p<0.001.

For the same practical and economic reasons, the random sample of 44 NC patients was used in the cytokine analysis of plasma from SLA-stimulated whole blood. Compared to the plasma from the NC patients, that of NH-AIRL patients contained more IFN-γ (p<0.001), TNF-α (p<0.050), granzyme B (p<0.001), IP-10 (p<0.001) and IL-2 (p<0.001) (Fig 3). The CVL patients showed the same pattern. Compared to the CVL patients, the AVL patients showed lower values for IFN-γ (p<0.050), IP-10 and IL-2 (p<0.010) (Fig 3).

### Cytokine profiles from PHA-stimulated samples

Non-specific stimulation of the PBMC with PHA-M led to an efficient Th1 response that was similar in the NC, AIRL and CVL subjects; not for AVL, as expected (S3 Table). Granzyme B production in PHA-stimulated PBMC cultures was however, significantly higher in the CVL subjects than in the NH subjects (p<0.050). In addition, IP-10 and IL-2 production in PHA-stimulated blood were significantly higher in the NH-AIRL subjects than in the CVL subjects (p<0.050).

### Identification of cured status and asymptomatic infection

The optimal cytokine cut-off values indicating cured status and asymptomatic infection were determined by means of ROC analyses (S4 and S5 Tables). Table 2 shows the proportion of patients with cytokine levels above their respective cut-off values.

**Table 2:** Proportion of patients that produced cytokine levels above the calculated optimal cut-off values after SLA-stimulation of a whole blood sample. *NH-AIRL* asymptomatic immune responders to *Leishmania*, *CVL* patients cured of leishmaniasis.

	Supernatants of SLA-stimulated PBMC cultures			Plasma from SLA-stimulated whole blood	
Analytes	NH-AIRL	CVL	Analytes	NH-AIRL	CVL
	N (%)	N (%)		N (%)	N (%)
IFN-γ	20/20 (100)	13/14 (92)	IFN-γ	15/20 (75)	10/11 (90)
TNF-α	17/20 (85)	9/14 (64)	TNF-α	9/20 (45)	7/11 (63)
Granzyme B	18/20 (90)	13/14 (92)	Granzyme B	14/20 (70)	8/11 (72)
IP-10	15/20 (75)	10/14 (71)	IP-10	18/20 (90)	10/11 (90)
IL-2	0/20 (0)	0/14 (0)	IL-2	20/20 (100)	9/11 (81)

The NH-AIRL patients showed the ability to mount a specific Th1 response against *Leishmania*. After the stimulation of their PBMC, IFN-γ was produced above the cut-off, identifying 100% of this population. In plasma from SLA-stimulated whole blood, the production of IP-10 identified 90% of the patients with asymptomatic infection, but as described in immunocompetent subjects [10], IL-2 production identified 100% of this population. Of note, TNF-α production may be influenced by the presence of anti-TNF treatments in the blood of some patients (not critical in washed PBMC).

## Discussion

The present work shows that cytokine assays can be used to detect asymptomatic *Leishmania* infection in patients receiving immunosuppressant treatment for autoimmune conditions, and to detect cure in those who require treatment for VL. In an earlier study, our group showed that cytokine release assays can be used to determine cured status in patients who have undergone solid organ transplantation [12]. In a further study in patients coinfected with VIH/*Leishmania*, we showed the usefulness of the specific lymphoproliferative response to SLA as a marker of cure in these patients, and as an indicator of the possibility of withdrawing secondary prophylaxis [10].

In the present work, the CVL patients showed a specific cellular response and the strong production of Th1-type cytokines. It remains unknown, however, how long these patients maintain the immunological memory developed, or how this might be related to relapse. New studies over longer periods will be needed to determine this. Certainly, these patients produced IFN-γ, granzyme B and IP-10 at levels allowing them to be biomarkers of cure. These cytokines should therefore be monitored as part of these patients’ management.

Molecular and serological techniques are very useful to diagnose AVL in autoimmune patients, mainly ELISA /IFAT and PCR in bone marrow [16]. As the present results show, they have certain limitations in determining VL cure. Although Ln-PCR did detect the parasites during active VL [16], the number present in the blood declines rapidly to undetectable levels after the first treatment dose [16]. This does not, however, indicate a cure to have been achieved; some parasites may remain in the bone marrow. In previous work [17], the serological tests used (the same as in the present study) were clearly less sensitive as a diagnostic tool in patients coinfected with HIV/*Leishmania* since >40% made no antibodies. In contrast, in patients immunosuppressed due to their having undergone solid organ transplantation, its sensitivity was found to be about 93% [18]. It has also demonstrated to be a sensitive tool for use in patients receiving anti-TNF treatment [16,19]. However, in the present patients, the serological tests continued to return positive results after cure, rendering them unsuitable for determining when cure is actually achieved.

An asymptomatic person is usually regarded as someone from an endemic area who shows an immune response (either antibodies or a specific cellular response) against *Leishmania*, or who has parasites—or parasite DNA—in the blood [20–22]). Molecular methods such as PCR have been used less often to identify asymptomatic infection due to low parasitemia [23]. Serological markers are often measured but they can revert to negative within 4 months [24]. However, cell immunity usually remains positive for several years, sometimes even throughout an individual’s life [25], and this is the main reason to use tests that detect the cell-mediated immune response when screening for asymptomatic subjects in areas with endemic leishmaniasis. In fact, a cross-sectional survey of the immunocompetent population living in the same area (Fuenlabrada, Madrid, Spain) recently showed a high prevalence of asymptomatic subjects measured by WBA and IL-2 detection (20.7%) compared to the low levels detected by serological techniques (1.1%) [26].

By using these assays, the present results also show that some 21.27% of the NH patients had been in contact with the parasite. Supernatants from SLA-stimulated PBMC cultures (from CPA tests) can be used to look for IFN-γ, while SLA-stimulated plasma (from WBA tests) can be used to look for IP-10 and IL-2, all of which are biomarkers of the asymptomatic condition. However, preparing the latter for cytokine levels to be determined is much easier; indeed, WBA tests hold much promise in the point-of-care setting. Immunosuppressed but asymptomatic immune responders to *Leishmania* should be monitored to help prevent the development of VL and to treat it early if it does appear. This need is further to that outlined in a study of HIV+ and transplanted patients from the same area, among whom similar asymptomatic individuals were detected [10,12].

The present work is the first to show that the elevated production of certain cytokines following the SLA-stimulation of whole blood can be used to identify asymptomatic *Leishmania* infection in immunosuppressed patients, and to confirm cure from VL in those who developed this disease. Earlier studies in immunocompetent subjects showed the importance of IFN-γ, IL-2 and IP-10 in the identification of asymptomatic immune responders to *Leishmania* (NH-AIRL-type patients) [9,27]. These same molecules have also been shown useful in the detection of asymptomatic *Leishmania* infection in transplanted patients and patients coinfected with HIV [11,12].

Although some unspecific reactions have been described for SLA antigen in patients with tuberculosis, brucellosis, typhoid fever, malaria or trypanosomiasis, none of our patients had had previously any of these infections. In addition, our previous works with solid organ transplant patients reinforce the specificity of the antigen in patients under immunosuppressive treatments. Although current literature has not shown unspecific cytokine release for SLA antigen in blood from autoimmune patients, molecular mimicry exists between antigens from *Leishmania* sp. and patients with systemic lupus erythematosus [28], consequently new studies are need in order to clarify this complex issue.

The main limitation of this study is the size of the cohorts of immunosuppressed patients because the heterogeneity of the treatments and patients’ conditions could affect the obtained results. In addition, other epidemiological areas should be also studied.

In conclusion, the present results show that cytokine release assays can be used to confirm the cure of VL in patients with autoimmune disease, in whom immunosuppressive treatment is usually suspended; confirmation of a cure would allow immunosuppression to be re-established at the earliest opportunity. These assays can also be used to identify patients with autoimmune disease who have asymptomatic *Leishmania* infection, and who are therefore at risk of developing VL. Such knowledge would also be useful in efforts to control the spread of *Leishmania*.

## Supporting information

S1 Table Characteristics of the no history of leishmaniasis subjects (NH), normal controls (NC), asymptomatic immune responders to *Leishmania* (*NH-AIRL)*. (XLSX)

S2 Table Characteristics of the 14 patients ostensibly cured of VL. (XLSX)

S3 Table Cytokines’ production after PHA-M stimulation of PBMC or whole blood from patients under immunosuppressive treatment for autoimmune disease. Median and IQR are shown for the subjects without history of leishmaniasis (NH), asymptomatic immune responders to *Leishmania* (NH-AIRL), patients cured of VL (CVL) and patients with active VL (VL).
(XLSX)

S4 Table Accuracy of detection of CVL patients and asymptomatic subjects via cytokine analysis of SLA-stimulated PBMC cultures. (XLSX)

S5 Table Accuracy of detection of CVL patients and asymptomatic subjects via cytokine analysis of SLA-stimulated plasma from the whole blood assay. (XLSX)
